# Characterization of the Asian Citrus Psyllid-‘*Candidatus* Liberibacter Asiaticus’ Pathosystem in Saudi Arabia Reveals Two Predominant CLas Lineages and One Asian Citrus Psyllid Vector Haplotype

**DOI:** 10.3390/microorganisms10101991

**Published:** 2022-10-08

**Authors:** Yasser E. Ibrahim, Jorge R. Paredes-Montero, Mohammed A. Al-Saleh, Arya Widyawan, Ruifeng He, Mahmoud H. El Komy, Hathal M. Al Dhafer, Noel Kitchen, David R. Gang, Judith K. Brown

**Affiliations:** 1 Plant Protection Department, College of Food and Agriculture Sciences, King Saud University, Riyadh 11451, Saudi Arabia; 2 School of Plant Sciences, The University of Arizona, Tucson, AZ 85721, USA; 3 Department of Biology, Saginaw Valley State University, Saginaw, MI 48710, USA; 4 Facultad de Ciencias de la Vida, Escuela Superior Politécnica del Litoral, ESPOL, Campus Gustavo Galindo Km 30.5 Vía Perimetral, P.O. Box 09-01-5863, Guayaquil 090902, Ecuador; 5 Institute of Biological Chemistry, Washington State University, Pullman, WA 99164, USA

**Keywords:** citrus greening disease, invasive species, phylogeography, prophage genome sequencing

## Abstract

In Saudi Arabia (SA), the citrus greening disease is caused by ‘*Candidatus* Liberibacter asiaticus’ (CLas) transmitted by the Asian citrus psyllid (ACP) *Diaphorina citri*. The origin and route(s) of the ACP-CLas pathosystem invasion in SA have not been studied. Adult ACP were collected from citrus trees in SA and differentiated by analysis of the mitochondrial cytochrome oxidase I (*mtCOI*) and nuclear copper transporting protein (*atox1*) genes. A phylogenetic analysis of the *Wolbachia* spp. surface protein (*wsp*) gene was used to identify the ACP-associated *Wolbachia* spp. A phylogenetic analysis of the *atox1* and *mtCOI* gene sequences revealed one predominant ACP haplotype most closely related to the Indian subcontinent founder populations. The detection and identification of CLas in citrus trees were carried out by polymerase chain reaction (PCR) amplification and sequencing of the *16S rDNA* gene. The CLas-integrated prophage genomes were sequenced, annotated, and used to differentiate CLas populations. The ML and ASTRAL trees reconstructed with prophages type 1 and 2 genome sequences, separately and concatenated, resolved two major lineages, CLas-1 and -2. The CLas-1 clade, reported here for the first time, consisted of isolates from SA isolates and Pakistan. The CLas-2 sequences formed two groups, CLas-2-1 and -2-2, previously the ‘Asiatic’ and ‘Floridian’ strains, respectively. Members of CLas-2-1 originated from Southeast Asia, the USA, and other worldwide locations, while CLas-2-2 was identified only in Florida. This study provides the first snapshot into the status of the ACP-CLas pathosystem in SA. In addition, the results provide new insights into the pathosystem coevolution and global invasion histories of two ACP-CLas lineages with a predicted center of origin in South and Southeast Asia, respectively.

## 1. Introduction

The Asian citrus psyllid (ACP) *Diaphorina citri* (Kuwayama, 1908) (Hemiptera: Liviidae) is the insect vector of the fastidious bacterial pathogen ‘*Candidatus* Liberibacter asiaticus’ (CLas) [1,2] and the causal agent of Huanglongbing (HLB), also known as citrus greening (CG) disease, with a hypothesized origin in Southeast Asia [3]. Citrus greening is among the most important diseases of citrus trees worldwide. Infected trees exhibit reduced fruit quality and yield, branch die-back, and, eventually, cause tree death [4,5]. The ACP was described in 1908 in Taiwan [6], and the suspect bacterial causal agent CLas was first reported in Southern (mainland) China in 1919 [7]. During 2000–2010, ACP-CLas was introduced into most citrus-growing countries of Asia, Africa, the Americas, and the Middle East [8,9,10,11].

In Saudi Arabia, ACP was first identified in the early 1970s [8,9] and has since become established in the western and coastal micro-niches [8]. The dispersal of ACP in Saudi Arabia citrus groves may have been facilitated by the importation of and/or distribution of locally infected citrus budwood and rootstock seedlings [1]. Greening-like symptoms were first observed in Saudi Arabia and North Yemen [5] in the mid-1980s. The suspect causal agent was identified as CLas by electron microscopy, confirming its distribution in the Bisha Khurman, Taif, Turabah, and Zaymah regions [9]. The effects of the ACP-CLas pathosystem in these regions of Saudi Arabia led to the replacement of sweet orange and mandarin crops with Mexican lime, a citrus hybrid that is less susceptible to CLas [9]. Four decades later, the invasion route(s) of the ACP-CLas pathosystem in Saudi Arabia has remained undetermined. The knowledge of such pathways can contribute valuably to the risk assessment and prevention of new introductions and outbreaks.

The high resolution of mitochondrial gene sequences at the microevolutionary level is attributable to naturally occurring substitutions in the mitochondrial genome, which accumulate at a much faster rate than the nuclear genome counterpart [12]. The evolutionary informativeness of the mitochondria cytochrome oxidase I (*mtCOI*) gene has been demonstrated for tracing the endemism of insect pests and vector species [13,14,15,16,17] and for predicting the invasion route(s) of species introductions [18,19,20,21]. A previous analysis of the ACP *mtCOI* gene resolved two main ACP lineages associated with ‘hotspots’ of endemism recognized in South and Southeast Asia, respectively [18,21,22,23,24,25]. The *Antioxidant 1 Copper Chaperone* (*atox1*) protein [26] is an informative molecular marker for differentiating geographically isolated intraspecific ACP populations, including those from China and Pakistan [24]. This marker has enabled the predictions of ACP origins by alignment and phylogenetic analysis of the *atox1* reference sequences available in the GenBank database. Based on the propensity for the horizontal transfer of facultative bacteria in the genus *Wolbachia* among related and divergent insect hosts [27,28,29,30], informative molecular genetic markers from *Wolbachia* spp., such as the *wsp* gene sequence, are often indicative of recent insect origins and, therefore, potentially, of recent invasion histories [31]. In particular, this molecular marker may be informative for tracing interactions between ACP populations that coexist or have lived in sympatry or relatively close proximity [21]. Thus, comparisons of intraspecific divergences among ACP populations in Saudi Arabia using informative molecular genetic markers are expected to be informative for determining the origins of founder populations and, thereby, identifying potential entry routes.

The detection of CLas using polymerase chain reaction (PCR) amplification of the highly conserved bacterial 16S ribosomal RNA (16S rRNA) gene [32] has become routine; however, the extensive conservation in the 16S rRNA gene does not permit phylogeographical or biological differentiation [33,34]. In contrast, prophages have been shown to resolve genome-specific diversity, which can be phylogenetically informative [35,36,37]. Three predominant prophage types were associated with CLas, initially: SC1, SC2, and P-JXGC-3, corresponding to Types 1, 2, and 3, respectively [36,38,39,40,41,42]. Prophage genome-associated polymorphisms have led to the identification of potentially informative regions that are phylogeographically informative [35,39,41]. The first complete genome of the prophage (type 1) was determined for CLas strain psy62 infecting ACP in Florida, USA [43], while type 1 and 2 genome sequences were determined from the CLas UF506 isolate experimentally inoculated periwinkle plants [36]. A phylogenetic analysis of *terL*, which encodes the prophage terminase gene and prophage type 1 and 2 whole-genome sequences, resolved two CLas geographic subtypes (or possibly strains) extant in the USA. One subtype has been shown to occur only in Florida (USA) (Floridian strain), while the other subtype is most closely related to previously reported isolates from Asia (Asiatic strain), respectively [39]. Additionally, the analysis of CLas-chromosome ‘tandem repeat number’ (*trn*) and ‘single nucleotide polymorphism 1′ (*snp1*) loci from infected citrus trees in California [44,45] resulted in the identification of two phylogeographical variants (or possibly strains) that are most closely related to isolates from the Americas and China [39,46], which suggests that at least two different CLas introductions have occurred in California (USA).

In this study, the genetic variability, demography, and invasion history of the ACP-CLas pathosystem in Saudi Arabia were characterized by phylogenetic analysis of the ACP *atox1* nuclear gene and *mtCOI* gene fragments [24], the phylogenetically informative endosymbiont *Wolbachia* spp. Surface protein (*wsp*) [2,27,47], and CLas-associated full-length prophage genome regions [37,41]. These results provide the first snapshot of the recent invasion history of the ACP-CLas pathosystem in Saudi Arabia.

## 2. Materials and Methods

### 2.1. Asian Citrus Psyllid Samples and DNA Extraction

Adult ACP for molecular marker and prophage analyses were collected from citrus trees using a hand-held aspirator, placed live in 95% ethanol, and stored at −20 °C. Citrus leaf samples for CLas detection were collected from symptomatic trees, immediately frozen, and stored at −20 °C until processing. Psyllid [12] and leaf [31] samples were collected from sweet orange (*Citrus sinensis*), Mandarin (*C. reticulata*), and Mexican lime (*C. aurantiifolia*) in the four major citrus-growing regions of Saudi Arabia, Al Baha, Jizan, Makkah, and Najran (Supplementary Table S1).

Total DNA was extracted from individual field-collected adult ACP using a CTAB (100 mmol/L Tris-HCl pH 8.0, 20 mmol/L EDTA pH 8.0, and 1.4 mol/L NaCl containing 0.2% 2-mercaptoethanol and 2% hexadecyltrimethylammonium bromide) lysis buffer. Individual adult psyllids (ACP) were transferred to a 1.5 uL Eppendorf tube containing 20 2mm zirconium beads in 600 uL of CTAB lysis buffer. Psyllids were homogenized using a Storm 24 Bullet Homogenizer (Stellar Scientific, Baltimore, MD, USA). Homogenates were transferred to a sterile Eppendorf tube containing an equal vol of chloroform, and centrifugation was carried out at 16,000× *g* for 3 min. The supernatant was transferred to a sterile 1.5 uL Eppendorf tube. One vol isopropanol was added, and the samples were held at -20 C for 2 h. The total nucleic acids were collected by centrifugation at 16,000× *g* for 15 min, washed with 70% ethanol, and air-dried at room temperature. The pellet was dissolved in 10 mmol/L Tris-HCl, pH 8.0.

### 2.2. Amplification and Sequencing of ACP mtCOI, Nuclear atox1, and Wolbachia Genes

The *mtCOI* gene fragment was amplified by polymerase chain reaction (PCR) using the primers *COI*-F-5′-GGTCAACAAATCATAAAGATATTGG and *COI*-R-5′-TAAACTTCAGGGTGACCAAAAAATCA [24] that yielded an amplicon of 834 bp. The cycling parameters consisted of 30 cycles of denaturation at 95 °C for 15 s, annealing at 57 °C for 15 s, and elongation at 72 °C for 50 s. For the nuclear gene *atox1*, a 750-bp fragment was obtained by PCR amplification using the primers Cutp-F-5′-CTGTGCTCCCTGTTCTAAG and Cutp-R-5′-ATACTCATTAGTTGAGGGTAG [21]. The cycling conditions consisted of 30 cycles of denaturation at 95 °C for 15 s, annealing at 52 °C for 15 s, and elongation at 72 °C for 50 s. The *Wolbachia* spp. *wsp* gene fragment was amplified by PCR using the primers *wsp*–81F-5′-TGGTCCAATAAGTGATGAAGAAAC and *wsp*–691R5′-AAAAATTAAACGCTACTCCA to obtain the expected size product of 450 bp [21]. The cycling parameters consisted of 30 cycles of denaturation at 95 °C for 15 s, annealing at 55 °C for 15 s, and elongation at 72 °C for 60 s. Each PCR reaction consisted of 1X JumpStart REDTaq ReadyMix (Sigma-Aldrich, St. Louis, MI, USA), oligonucleotide primers (0.4 μmol/L each), 20 ng of psyllid DNA, and double-distilled (dd) water.

### 2.3. ‘Candidatus Liberibacter Asiaticus’ Detection in Citrus Leaf Samples

Total DNA was isolated from citrus leaf samples (Mexican lime, mandarin, and sweet orange) from the different citrus-growing regions of Saudi Arabia. Detection of CLas in the leaf samples was carried out by quantitative polymerase chain reaction (PCR) amplification of the CLas 16S rRNA gene [32]. Samples yielding a Cq value of < 29 (*n* = 30) were verified by Sanger sequencing, following PCR amplification of an 853-bp fragment of the 16S rRNA gene using primers CLas16S-F-5′-GGTGAAATCCCAGGGCTCAA and CLas16S-R-5′-ACCGTCTTCAGGCAAAACCA [24]. The cycling parameters consisted of 30 cycles of denaturation at 95 °C for 15 s, annealing at 59 °C for 15 s, and elongation at 72 °C for 50 s. Amplicons of the expected size were ligated into the pCR2.1 plasmid vector (Thermo-Fisher Scientific, Waltham, MA, USA) and transformed into *E. coli* competent cells by heat shock transformation according to the standard molecular biology protocols. Three to five colonies per sample were screened by colony PCR with the M13 universal primers. The plasmid vector harboring an insert (amplicon) of the expected size was confirmed following agarose gel electrophoresis (1%; Tris–Acetate–EDTA buffer, pH 8.0). The plasmid vector was purified and subjected to bidirectional Sanger sequencing. CLas-infected leaf samples of one isolate each from Florida and the Washington State greenhouse-maintained CLas-citrus cultures were included as positive CLas controls.

### 2.4. Phylogeographic Analyses of the Asian Citrus Psyllid Mitochondrial and Nuclear Gene and Wolbachia Wsp Gene Sequences

Reference sequences for all molecular markers were downloaded from the National Center for Biotechnology and Information (NCBI) repository. The sequences determined in this study were aligned with reference sequences using Muscle v3.8.425 software, implemented in Geneious Prime software v2021.1.1 (Biomatters, Ltd. Auckland, New Zealand). The alignment was trimmed to remove the poor-quality ends and short sequences. Single nucleotide polymorphisms were identified using the “Find variations/SNPs” function available in the “Annotate and Predict” menu implemented in Geneious Prime v2021.1.1 (Biomatters, Ltd. New Zealand). Maximum likelihood phylogenetic trees were reconstructed using the RaxML algorithm with the GTR+GAMMA nucleotide model of the evolution for 100 bootstrap replicates [48]. The *D. lycii* sequence was used to root the *mtCOI* tree. The nuclear *atox1* and the *wsp* alignments were midpoint-rooted, because no suitable reference sequence was available for either molecular marker. Geographic coordinates for ACP collections and reference sequences were used to plot the spatial distribution of ACP populations and associated microbiota using the “Biological Records Tools” implemented in QGIS v3.16.3 software (https://qgis.org/en/site/ accessed on 15 May 2022). The geographic information provided with the GenBank references was downloaded using a custom python script.

### 2.5. Agilent SureSelect XT HS Target Enrichment Library Construction and Sequencing

Agilent enrichment libraries were constructed for sixteen CLas-positive samples, each having a Ct value of < 29. Libraries were sequenced using the Illumina HiSeqX Ten platform, which generated 148 GB of data consisting of 982 million reads with an average Q30 quality of 92% (S10). The Illumina read ends were edited using Trimmomatic v0.32 [49]. High-quality reads were mapped to the prophage reference SC1 genome sequences (Accession NC_019549) and SC2 (Accession NC_019550), respectively, using Bowtie 2 v2.4.5 software and default parameters [50]. The consensus sequences were determined using the *mpileup*, *call,* and *consensus* functions available in BCFtools v1.15.1 [51].

### 2.6. Prophages Phylogenetic Analyses

The prophage consensus sequences determined here were aligned using the FFT-NS-1 algorithm available in MAFFT v7.450 software [52]. The type 1 prophage sequences of approximately 38 Kbp in size were aligned with GenBank references from China (CP004005), Colombia (CP054558), Florida (CP001677, HQ377374), Pakistan (MT899444), and Reunion Island (CP061535). The type 2 prophage sequences of approximately 37 Kbp were aligned with reference sequences determined from ACP-prophage populations from California (CP029348); China (CP004005, CP019958, CP010804, and CP045565); Colombia (CP0545580); Florida (CP040636, CP001677, HQ377374, and JX275492); Reunion Island (CP061535); and Thailand (CP041385). The maximum likelihood (ML) analysis was carried out using RAxML v 8.2.11 with a GTR + G model of evolution and 100 rapid bootstrap replicates [48] for the individual prophage sequence and the prophage types 1 and 2 concatenated sequences.

Prophage types 1 and 2 were annotated using the NC_019549 and NC_019550 references, respectively. The prophage markers used in the phylogenetic analysis were selected from among the annotated aligned genome sequences determined here and based on the respective homologs also occurring in the ‘reference SC1 and SC2 prophage genome’ sequences (see the accession numbers above) available in the GenBank database (note: many available types 1 and 2 phage genome sequences are incomplete). Six type 1 and ten type 2 informative loci all available from GenBank and those determined in this study were used to reconstruct the phylogenetic tree with RaxML, as described. The optimal model of molecular evolution was determined for each gene using PartitionFinder v2.1.1 [53]. The individual gene trees were summarized by a coalescent analysis using ASTRAL v 5.5.9 [54] with the default parameters, and the clades were statistically supported by posterior probabilities.

## 3. Results

### 3.1. Phylogeographic Analysis of the Asian Citrus Psyllid mtCOI Sequence

**Figure 1:**
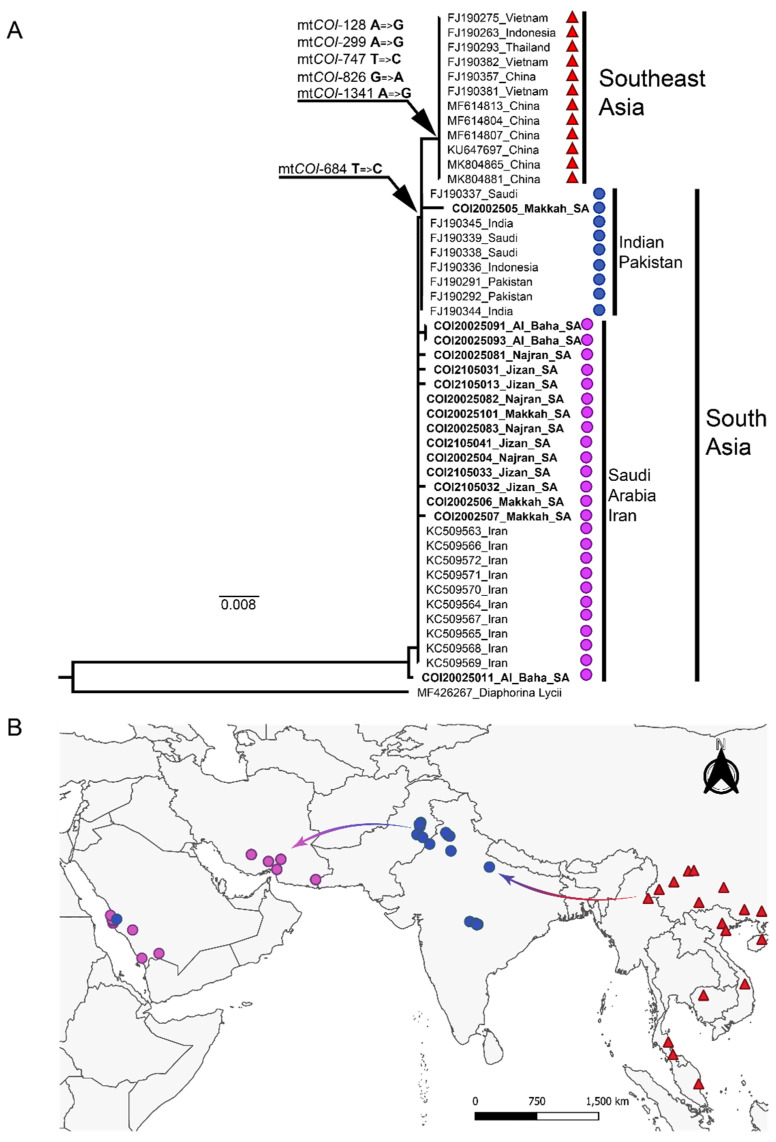
Phylogeny based on single nucleotide polymorphisms or character states in relation to and geographical distribution of Asian citrus psyllid (ACP) haplotypes in Asia and the Middle East. The phylogeographic breaks (**A**) are consistent with geographical isolation (**B**) between haplotypes. The predicted center of origin for ACP is Southeast Asia, where citrus species have also originated, based on genetic analyses and fossil records [56]. Arrows show the inferred hypothetical ancient route of ACP migration leading to the speciation of the phylogeographic haplotypes analyzed here.

Based on the *mtCOI* partial sequence (Figure 1), the ACP collected in Saudi Arabia and ACP reference sequences from Iran were nearly identical and exhibited an extremely low intra-haplotypic nucleotide divergence at 0.006%. The ACP populations from Saudi Arabia and Iran harbored a thiamine residue at position 684 of the *mtCOI*, a SNP that occurred consistently among all of the *mtCOI* sequences available for this haplotype (Figure 1). The Saudi Arabian populations were most closely related to members of a clade extant in Pakistan and India, one of two recognized geographical subcenters of ACP origin. In contrast, haplotypes from the Indian subcontinent harbored one SNP consisting of a cytosine residue (instead of thiamine) at position 684. For one sample, *COI*2002505 (Makkah region) and the reference sequences FJ190337-39 from Saudi Arabia, the *mtCOI* sequences were identical to those of the Indian subcontinent haplotype harboring the fixed mt*COI*-684 cytosine. These patterns were considered suggestive of one or more recent introductions in Saudi Arabia.

Reference *mtCOI* sequences from Southeast Asia and China corresponded to a haplotype that was distinct from those extant in Pakistan/Indian Subcontinent and Saudi Arabia at 0.5 and 0.7% nt divergence, respectively. Unique SNPs were identified in the Southeast Asia and China *mtCOI* sequences, which differentiated them from haplotypes in Iran and Saudi Arabia. The fixed cytosine residue identified in the Indian subcontinent haplotypes also occurred in the Southeast Asia haplotype, which additionally harbored five silent mutations at positions 128, 299, 747, 826, and 1341. The Southeast Asia haplotype is a predicted introduction in Pakistan and is known to be the predominant ACP haplotype in South American citrus plants [18,55].

### 3.2. Phylogenetic Analysis of the Asian Citrus Psyllid atox1 Nuclear Sequence

**Figure 2:**
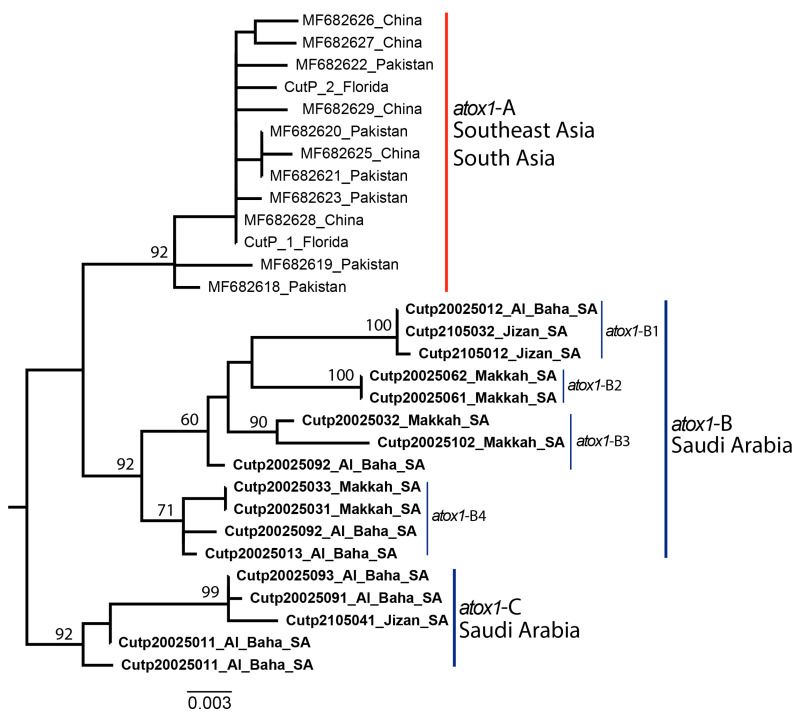
Phylogeny of Asian citrus psyllid populations based on the nuclear gene *atox1*. The psyllid collections from Saudi Arabia formed two clades based on the *atox*1-B and *atox1*-C sequences, with *atox1*-B containing four variants, *atox1*-B1–4 (blue). The nearly globally distributed ‘invasive’ ACP was represented by one nuclear haplotype, *atox1*-A, consisting of populations from China and Pakistan (red).

Three phylogenetic groups, *atox1*-A, -B, and -C, were resolved by phylogenetic analysis of the *atox1* gene (Figure 2). The nucleotide polymorphisms at positions 321 and 460 in the *atox1*gene separated *atox1*-A from *atox1*-B and -C, supporting the long-term isolation between the ACP population in Saudi Arabia and Iran from populations in the Indian subcontinent and China. The *atox1*-A cluster, at 0.6% intra-clade divergence, consisted of samples from China and locations in Pakistan where the ACP (Southeast Asia Lineage) was thought to have been introduced. The Florida ACP collections, which were grouped in the South Asia lineage (Indian subcontinent), also clustered with the *atox1*-A clade, a pattern suggestive of an ongoing intraspecific gene flow where both ACP major lineages coexist, apparently, near the boundaries of their respective natural niches. *atox1*-B, at 1.6% intra-clade divergence, was the most genetically variable group among the *atox1* clusters and consisted of four *atox1*-B haplotypes known to occur only in Saudi Arabia, *atox1*-B1–4. Haplotype *atox1*-B1 had a fixed guanine residue at positions 435 and 754, respectively, and an adenine residue at position 686. In contrast, the *atox1*-B2 and -B3 haplotypes had fixed guanine and cytosine residues at positions 50 and 65, respectively. Further, *atox1*-B3 differed from *atox1*-B2 by harboring cytosine, adenine, guanine, thymine, guanine, and adenine residues fixed at positions 336, 484, 497, 550, 602, and 628, respectively. Four nucleotide polymorphisms were characteristic of haplotype *atox1*-B4, with one thymine, adenine, adenine, and thymine residue each fixed at positions 374, 422, 438, and 656, respectively. Notably, the cluster *atox1*-C has not been previously recognized until this report and, thus far, has been found only in Saudi Arabia. The *atox1*-C cluster harbored four fixed character states, consisting of guanine, thymine, adenine, and thymine residues at positions 21, 23, 171, and 642, respectively, and their sequences exhibited a low intra-clade divergence of 0.8%, highly suggestive of the recent diversification.

### 3.3. Phylogenetic Analysis of Wolbachia spp. Wsp Sequences

**Figure 3:**
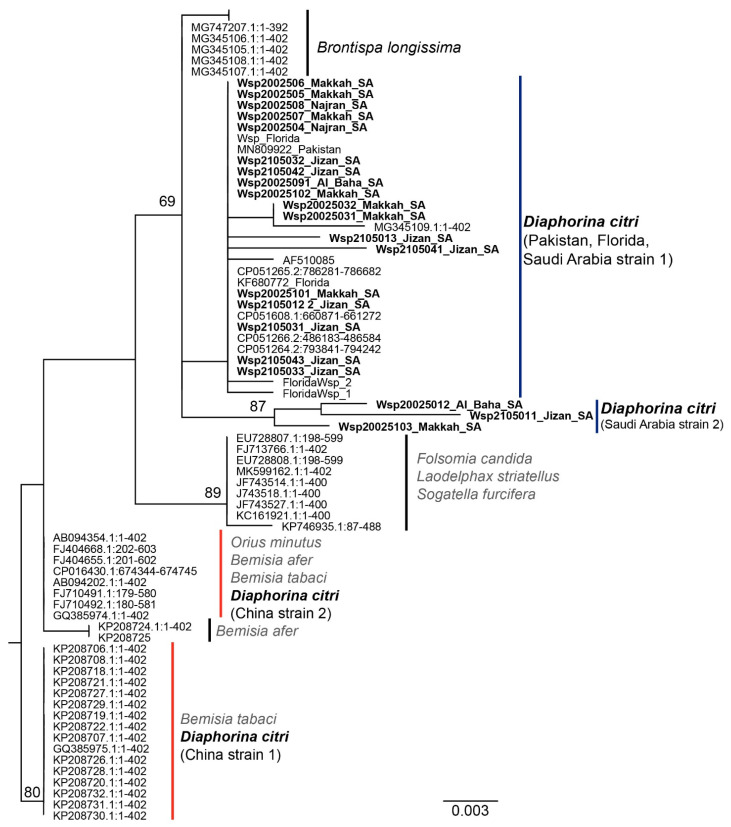
Phylogeny of the Wolbachia surface protein gene (wsp) associated with *Wolbachia* spp. endosymbionts of Asian citrus psyllid and representative insect species.

An analysis of the phylogenetically informative *wsp* sequence for the Saudi Arabian *Wolbachia* spp. isolates with *wsp* reference sequences available in GenBank indicated that at least two Wolbachia strains were associated with the ACP populations in China (Figure 3). One *Wolbachia* strain from China, herein China strain 1, had three cytosines at positions 209, 228, and 390, respectively, and guanine at position 236. The *Wolbachia* strain from China, herein China strain 2, harbored three cytosines located in the same positions as China strain 1, plus an adenine (instead of guanine) at position 236. Both of the ACP-*Wolbachia* strains identified from ACP in China have been reported from the whitefly species *Bemisia afer* and *B. tabaci* and from a whitefly predator, *Orius minutus* [57]. Based on the *wsp* sequence analysis, the ACP populations from Saudi Arabia harbored two strains of *Wolbachia* spp.; however, they diverged from the strains extant in China by 1.4%. The *Wolbachia* spp. Saudi Arabia strain 1 was the most widespread among the two strains identified in ACP collections from Pakistan and Saudi Arabia, with Saudi Arabian strain 1 *wsp* harboring a unique adenine residue at position 350. Occurrence of the Saudi Arabia strain *Wolbachia* spp. 2 was rare and, until this report, was identified only from the ACP from Saudi Arabia collected in this study. Saudi Arabia strain 2 *wsp* harbored a unique guanine residue at position 283. These observations suggest the possibility that *wsp* sequence divergence occurred after the introduction of ACP into Saudi Arabia in the 1970s. Alternatively, the ACP population may have acquired a new *Wolbachia* strain after becoming established there.

### 3.4. Analysis of ‘Candidatus Liberibacter Asiaticus’ 16S rDNA Sequence from Citrus Leaf Samples

The CLas 16S rDNA sequence (854 bp) amplicons provided evidence of CLas infection of most of the citrus trees sampled in Saudi Arabia (data not shown). Although an alignment of the forty-six cloned amplicons obtained from citrus leaf samples showed no evidence of phylogenetically informative sites among the CLas isolates, either one or two SNPs were identified among the CLas sequences (Accessions OP106889-92). The clone 148,168 from Saudi Arabia and reference Florida 1_1 shared two SNPs, located at nucleotide positions 68 and 753, respectively, whereas the ACP colony from Florida (maintained as a sub-colony) at Washington State University (WSU) (WSU_1) and the Pakistan reference MG855673 each harbored a SNP at nt position 316. In contrast, an insertion located at position 622 was identified in the CLas-16S haplotype sequences from China, an observation that was consistent with the hypothesis that ACP-CLas from the Indian subcontinent and Southeast Asia, respectively, evolved as two phylogeographic lineages (Supplementary Figure S1). The CLas-16S haplotype sequences from Saudi Arabia were most similar to the previously published Pakistan references consulted in this study (Supplementary Figure S1).

### 3.5. Phylogenetic Analysis of Full-Length CLas-Prophage Genome Sequences

The phylogenetic analysis of full-length prophage genome sequences indicated the presence of two different CLas lineages. One clade, referred to as lineage CLas-1, was identified for the first time. CLas-2 has been previously identified, with clusters with one of two known subtypes [38,44]—here, CLas-2-1 (Asia) and -2-2 (Florida), respectively. The isolates from Saudi Arabia harbored both type 1 and type 2 prophages (Supplementary File S1), which sets this result apart from all other previous studies [39,45]. The average mapping rates of the types 1 and 2 prophage genome sequences determined from CLas in collections from Saudi Arabia were 25.95% (Supplementary Table S2) and 30.69% (Supplementary Table S3), respectively, with the SC1 and SC2 reference sequences at 0.16–47.67% and 0.23–67.74% reads, respectively. The differences in mapping efficiencies may have resulted from different levels of CLas genome accumulation in citrus leaf samples collected in this study. The annotation of assembled prophage sequences identified 37–58 and 42–51 annotated SC1/type1 and SC2/type 2 proteins (Supplementary Tables S2 and S3), respectively. The number of predicted SC1/type 1 proteins was apparently constrained by the mapping rate potentially associated with the depth of reading coverage.

### 3.6. Phylogeny of CLas Reconstructed for the SC1/type 1 Prophage Full-Length Sequences

**Figure 4:**
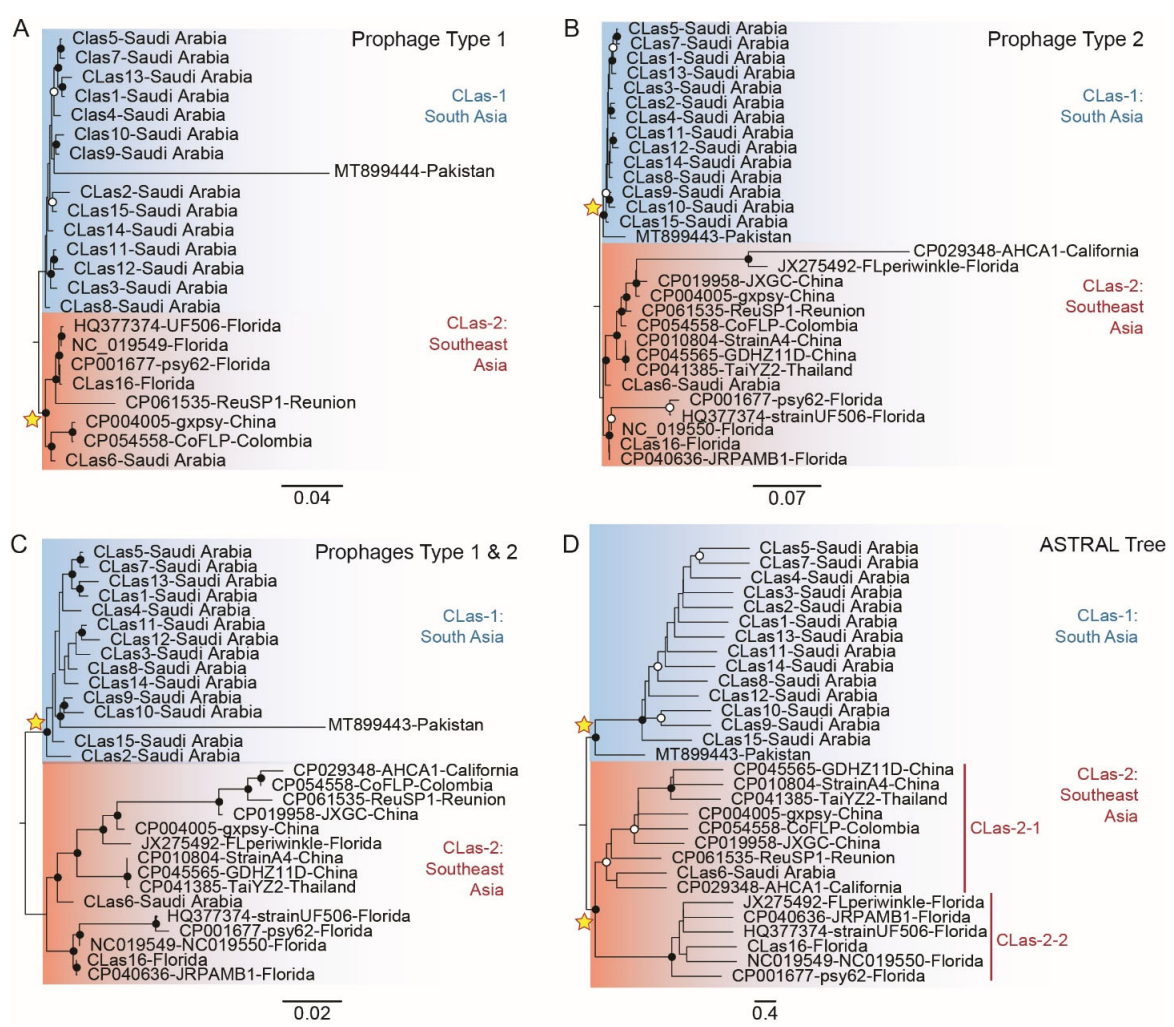
Phylogenetic relationships between ‘*Candidatus* Liberibacter asiaticus’ (CLas) by a maximum likelihood analysis of CLas-associated prophage type1 (**A**), -prophage type 2 (**B**), and types 1 and 2 concatenated full-length genome sequences (**C**). To reconcile the six SC1/type1 and ten SC2/type 2 tree topologies, a CLas species tree was reconstructed using ASTRAL (**D**). The single and concatenated CLas-prophage sequences are grouped as two clades, CLas-1 and CLas-2. The CLas-2 isolates formed two sister clades, CLas-2-1, which is widely distributed (‘Asiaticus’), and CLas-2-2, which occurs only in Florida (‘Floridian’). A star placed at major nodes indicates robust phylogenetic support.

A phylogenetic analysis of the full-length SC1/type1 prophage resolved two major CLas lineages, CLas-1 and -2 (Figure 4A). The CLas-1 lineage is reported in this study for the first time. The CLas-1 sequences were grouped as a paraphyletic clade consisting of 14 out of the 15 Saudi Arabian isolates whose closest relative was a type 1 prophage sequence from Pakistan. The Saudi Arabian CLas-1 isolates evolved at an average rate of 0.016 substitutions per site, whereas the Pakistan reference sequence exhibited a high mutation rate of 0.3681. The CLas-2 sequences formed a well-supported monophyletic clade (100% bootstrap value). The CLas-2 monophyletic lineage comprised the two subtypes/strains—here, CLas-2-1 and 2-2—also referred to as the ‘Asiatic’ and ‘Floridan’ strains, respectively, previously reported to be present in the USA [39,45]. CLas-2-1 (‘Asiatic strain) included isolates from China and other locations where CLas is known to be an introduced pathogen, including Colombia. CLas-2-2 (‘Floridian strain) included mainly the Florida (USA) isolate and an isolate from Reunion Island. The average number of substitutions per site for the CLas-2 lineage was faster than CLas-1 at 0.026, indicative of their relatively long-term isolation from founder CLas populations in Southeast Asia. One isolate from Saudi Arabia, ‘sample CLas6′, collected from a mandarin tree in urban Riyadh, was grouped with this ‘invasive’ CLas-2 lineage.

### 3.7. Phylogeny of CLas Reconstructed for the SC2/type 2 Prophage Full-Length Sequences

As was observed for the SC1/type 1 prophage, the phylogeny of full-length prophage SC2/type 2 resolved the two CLas lineages, CLas-1 and -2. In contrast, for the SC1/type 1 phylogeny, robust support was provided for the CLas-1 monophyly (100% bootstrap) comprising prophage isolates from Saudi Arabia and a prophage reference sequence associated with CLas in Pakistan. The type 2 isolates harbored an average number of substitutions per site of 0.014, which is similar to the substitution rate in type 1 prophage genomes. The mutation rate of the Pakistan type 2 reference prophage sequences was 16 times less compared to the type 1 prophage at 0.023 substitutions per site. CLas-2 consisted of two well-supported monophyletic subclades, herein CLas-2-1 and 2-2 (Figure. 4). One clade contained CLas-2-1, previously referred to as the ‘Asiatic’ strain [39,45], which has been identified in ACP from California (USA), China, Colombia, Florida (USA), Reunion Island, and Thailand, whereas CLas-2-2 grouped in clade 2 (referred to as the ‘Floridian’ strain) has been identified thus far only in Florida citrus trees. Based on the type 2 prophage genome, a CLas-2 genetic analysis indicated the genome diversification rapidly evolved faster than the CLas-1 lineage based on an average number of substitutions per site of 0.046.

### 3.8. Phylogeny of CLas Reconstructed from SC1/Type 1 and SC2/Type 2 Concatenated Genome Sequences

To remedy the discrepancies in bootstrap support of the CLas phage genome phylogenies, the two genome sequences were contented and used to reconstruct a partitioned tree (Figure 4C). The topology of the concatenated tree (Figure 4C) was indistinguishable from the CLas tree resolved for the prophage type 2 alone (Figure 4B). The concatenated tree had well-supported CLas-1 monophyly and a paraphyletic CLas-2 lineage containing subclades CLas-2-1 and -2-2, also referred to as the ‘Asiatic’ and ‘Floridian’ strains [39,45], respectively. The CLas-2-1 lineage consisted of Saudi Arabian isolates and the reference sequence from Pakistan. The CLas-2-2 clade consisted of sequences unique to Florida (‘Floridian’), while isolates from China, Thailand, and other locations were grouped in clade CLas-2-1. The latter CLas isolates represent exotic introductions, as well as the ‘CLas6′ isolated collected from an urban citrus tree in Saudi Arabia at some distance from commercial trees, a scenario that points to a second introduction.

### 3.9. Reconciled CLas Species Tree Given by SC1/Type 1 and SC2/Type 2 Orthologous Genes

The gene tree topologies were reconciled by constructing an ASTRAL species tree for six SC1/type1 and ten SC2/type 2CLas-prophage loci (Figure 4D). The ASTRAL tree was shown to support the monophyly of CLas-1 and 2 lineages with 91% posterior probability (pp), respectively. The CLas-1 lineage identified here for the first time were of Saudi Arabia sequences and a Pakistan reference sequence. Members of the CLas-2 monophyletic lineage are nearly globally distributed and grouped as two subtypes, herein CLas-2-1 and -2-1 strains ‘Asiatic’ and ‘Floridian’, respectively. The pp support for the CLas-2-1 ‘Asiatic’ clade was 77% compared to the CLas- 2-2 clade, which was well-supported, at 100% pp. The discovery of two distinct CLas lineages was consistent with the identification of two distinct ACP lineages with an origin in South and Southeast Asia, respectively.

## 4. Discussion

### 4.1. Invasion History of Asian Citrus Psyllid in Saudi Arabia

In this study, the collected ACP populations were from the Makkah, Al Baha, Jizan, and Nijran regions in Southern Saudi Arabia, where HLB were previously detected in citrus trees. Surprisingly, surveys for ACP did not reveal infestations in Al Qassim, Hail, Al Madina, Al Jouf, Tabuk, Asir, Riyadh, the Northern Borders, and the Eastern region. These observations suggested that ACP dispersal has apparently been limited to short-distance migration. Nonetheless, the movement of plant materials that may contain the ACP’s eggs, and nymphs may aid long-distance proliferation and establishment of the pest in new locations. Preventive measures such as surveillance, containment, and pest management are required to slow the spread of ACP to uninfected and HLB-free areas of Saudi Arabia. The dissemination range of adult psyllids has been reported to be 2 km within 12 days [58]. Thus, a southward dispersal is likely impending, making surveillance, containment, and pest management strategies essential to prevent ACP from expanding further northward into Saudi Arabia.

Even though *D. citri* is the most invasive pest on citrus, only several informative molecular markers, most commonly the mt-COI and -COII genes and cuticular protein genes, are available for analyzing genetic diversity and population structures [21,24,59]. Based on the mtCOI sequence analysis, the Saudi Arabia samples were nearly identical to sequences from Iran with an intra-haplotypic nucleotide divergence of 0.006%. Two divergent, predominant ACP lineages have been recognized based on the *mtCOI* gene, one that is endemic to Southeast Asia and another with a predicted origin in the Indian subcontinent (South Asia) [14,18,19,21,22]. Both lineages, therefore, have been introduced to locales far from their centers of origin. The ACP lineage from Southeast Asia is well-established in Brazil, whereas the lineage endemic to the Indian subcontinent has become the main ACP lineage in North America [18], including Florida, where ACP was first reported in the US [10]. The divergent phylogenetic relationships predicted for two main ACP clades suggest that psyllid populations in Iran and Saudi Arabia most probably originated from a founder population in the Indian subcontinent, indicating a historical westward expansion of ACP from one of its evolutionary origins. In addition, the *mtCOI* fragment for one ACP isolate from Makkah (Saudi Arabia) shared 100% nt identity with counterpart ACP from Pakistan and India, potentially indicative of nearly contemporaneous introductions. Further, the single nucleotide substitution at position 684 of the *mtCOI* gene from cytosine (C) to thiamine (T) suggested a relatively long-term geographic isolation between populations in Saudi Arabia and the Indian subcontinent. Given the relatively small sample size, it was not possible to determine whether the ancestral state of position 684 was a C or T residue. However, because one of the two recognized centers of origin of the genus, *Citrus* [56] and ACP [19] is in South Asia, and because haplotype diversity in the Indian Subcontinent is the highest among ACP populations in South and West Asia [22], populations from India and Pakistan would be expected to represent the ancestral state that is shared by populations in Saudi Arabian and Iran. Previous studies have shown that the Southeast ACP lineage occurs in Pakistan, an observation that suggests they represent introductions from China [21,24,59].

The ACP nuclear gene marker, *atox1,* resolved phylogenetic relationships that support the long-term isolation between the Iran-Saudi Arabia population and its founder ACP from the Indian subcontinent (South Asia) and ACP belonging to the Southeast lineage. However, some isolates from South and Southeast Asia, grouped within the *atox1*-A clade, with a low intra-clade divergence of 0.6%, suggesting potential ongoing intraspecific gene flow between the major ACP lineages where their natural niches overlap and where ACP has been introduced and become established. Although Saudi Arabia *mtCOI* sequences harbor a single nucleotide substitution, setting them apart from the South Asia ACP lineage originating on the Indian subcontinent, the observed 2.8% inter-clade divergence of nuclear *atox1* sequences points to the likelihood of recent geographic isolation resulting in limited gene flow between ACP in Saudi Arabia and its Indian Subcontinent and Southeast Asia counterparts.

The *wsp* gene of *Wolbachia* spp. was used as a phylogenetic marker to potentially inform the demographic and life history of ACP populations in Saudi Arabia. Based on the *wsp* sequence, nearly all ACP populations studied thus far harbor the same *Wolbachia* spp., suggesting an initial infection of ACP by this facultative endosymbiont that has been sustained for some time [29,60]. Here, two *Wolbachia* spp. were identified among ACP populations from Saudi Arabia. Strain 1 occurred in nearly all ACP from Saudi Arabia and Pakistan, while strain 2 was identified only in ACP from Saudi Arabia (Figure 3). Identification of a *Wolbachia* spp. associated with ACP from Saudi Arabia and Pakistan (Saudi Arabia strain 1, Figure 3) support the hypothesis that ACP from Saudi Arabia and Pakistan are most closely related among known ACP haplotypes. The presence of a second *Wolbachia* spp. associated with the Saudi Arabian ACP (Saudi Arabia strain 2, Figure 3) and its absence in ACP from Pakistan corroborate the hypothesis for recent isolation between the two populations since the initial founder event. Two other *Wolbachia* spp. (China strain 1 and 2, Figure 3) have been identified in ACP reference populations from China (Southeast lineage). Such *Wolbachia* spp. have been previously identified in two whitefly species, *Bemisia afer* and *B. tabaci*, and have been detected in the whitefly predator, *Orius minutus*.

### 4.2. Invasion History of ‘Candidatus Liberibacter Asiaticus’ in Saudi Arabia

Since the 16S rDNA sequence is highly conserved among CLas, it is not phylogenetically informative. However, one insertion (indel) at position 622 in China CLas isolates supported the ongoing speciation of CLas that mirrored ACP vector populations (Supplementary Figure S1).

Prophages are one of the most dynamic components of the bacterial genome. They have a numerous impact on host evolution and intraspecies variability [61]. Here, the genetic structure of CLas isolated from citrus cultivars sampled in the different regions in Saudi Arabia is reported for the first time. To investigate the CLas invasion history, the genome sequences were determined for CLas-associated prophages type 1 and 2. The CLas from Saudi Arabia harbored prophage SC1/types 1 and SC2/type 2, which is consistent with types 1 and 2 being the most common among CLas isolates studied thus far [37,38,39,40,41]. Alignment of the full-length SC1/type1, SC2/Type 2, and concatenated prophage genomes indicated that CLas isolates grouped phylogenetically as two major lineages CLas-1 and CLas-2. The CLas-1 lineage is reported in this study for the first time and consisted of 14 of 15 Saudi samples that were most closely linked to reference sequences from Pakistan. The CLas-2 lineage, putatively ‘invasive’, consisted of two subgroups, CLas-2-1 and -2-2, also referred to as the ‘Asiatic’ and ‘Florida’ strains, respectively. The CLas-2-1 has been associated with the CLas isolates extant in China, Colombia, Florida (USA), Reunion Island, and one Saudi Arabian sample collected from a mandarin tree in Riyadh, Saudi Arabia. The CLas-2-2 consisted of Florida isolates only. These results indicate that CLas-2 has undergone diversification more rapidly than CLas-1.

The ASTRAL tree provided support for the monophyly of both CLas lineages. In addition, CLas-2 populations clustered as two subgroups, CLas-2-1 and -2-2, which is consistent with previously reported strains ‘Asiatic’ and ‘Floridian’, respectively. The CLas-2-1 and 2-2 strains have been previously differentiated by a tandem repeat region (trn1), a single nucleotide polymorphism 1 (snp1), divergent prophage terminase (terL) sequences, and whole-genome prophages SC1 and SC2 [39,44,45]. The CLas-2.1 originates from Southeast Asia and is widely distributed throughout the world, whereas CLas-2-2 has been identified thus far only in Florida.

The association of two CLas groups, CLas-1 and -2, with one of two ACP lineages having a predicted center of origin in South and Southeast Asia, respectively, is suggestive of a robust co-evolutionary relationship between CLas and the ACP vector, a partnership that has been proposed to contribute to variability in CLas transmissibility and/or disease severity. Differences in symptom phenotype and virulence associated with the different CLas-prophage types have been proposed to influence disease severity in citrus trees and/or accumulation and/or transmission competency by the psyllid vector [62], and/or that prophages may provide protection against host reactive oxygen species (ROS) during CLas infection of citrus trees [63,64,65]. In CLas genomes, prophages may be absent [66], or occur singly [67]) or in pairs [36]. In southern China, 83% of CLas isolates harbored only prophage type 2 [38], while both 1 and 2 types have been detected in CLas in Pakistan [42]. However, the inability to detect prophage(s) from psyllid or plant samples may suggest the CLas titer is low, making phages undetectable [39]. In this study, prophage types 1 and 2 were associated with all of the CLas isolates sequenced from citrus samples collected in Saudi Arabia. The predicted occurrence of a major CLas variant or strain throughout Saudi Arabia, also identified in Pakistan, suggests that similar pathogen-host interactions and virulence characteristics are expected for CLas isolates occurring in both Saudi Arabia and Pakistan. The results of this study provide the first snapshot into the status of the ACP-CLas pathosystem in SA in relation to its center of origin in South Asia and contribute to a greater understanding of the distribution of CLas based on prophage genome signatures as potential indicators of CLas origin and potentially of CLas strain-specific disease severity.

## Data Availability

The sequences of the *mtCOI*, *atox1*, *Wolbachia* spp. *wsp*, and CLas *16S rRNA* genes were submitted to GenBank. The accession numbers are provided in Supplementary Table S1. The prophage data and sequences are provided as Supplementary files.

## Supplementary Materials

The following supporting information can be downloaded at https://www.mdpi.com/article/10.3390/microorganisms10101991/s1, Figure S1: Alignment of the ‘*Candidatus* Liberibacter asiaticus’ (CLas) 16S rDNA sequences. The small bars between the aligned sequences indicate locations of single nucleotide polymorphisms in the CLas-16S rRNA sequences; Table S1: Sample number, location, citrus species, and GenBank accession numbers for ‘*Candidatus* Liberibacter asiaticus’ and *Wolbachia* spp. surface protein gene (wsp) sequences amplified by a polymerase chain reaction from adult Asian citrus psyllid collected in Saudi Arabia; Table S2: Read mapping to the ‘*Candidatus* Liberibacter asiaticus’ Type 1 prophage of Saudi Arabian isolate genomes; Table S3: Read mapping to the ‘*Candidatus* Liberibacter asiaticus’ Type 2 prophage of Saudi Arabian isolate genomes; File S1: Concatenated SC1 and SC1 prophage genomes.
