# Supplementary material for: Characterization of the Asian Citrus Psyllid-‘Candidatus Liberibacter Asiaticus’ Pathosystem in Saudi Arabia Reveals Two Predominant CLas Lineages and One Asian Citrus Psyllid Vector Haplotype
Source: Microorganisms. 2022 Oct 8;10(10):1991. doi: 10.3390/microorganisms10101991 (PMC9610752; doi:10.3390/microorganisms10101991)
Supplement: Supplementary file 1 [file microorganisms-10-01991-s001.zip › Table S1.pdf]

Table S1. Sample number, location, citrus species, and GenBank accession numbers for '*Candidatus* Liberibacter asiaticus' and *Wolbachia* spp. surface protein gene (*wsp*) sequences amplified by a polymerase chain reaction from adult Asian citrus psyllid collected in Saudi Arabia.

**Sample #**	**Location**	**Citrus species**	**Mitochondria *COI* gene**	**GenBank Accession no.**	**Nuclear ATOX1 gene**	**GenBank Accession no.**	***Wolbachia* spp. *Wsp* gene**	**GenBank Accession no.**
**21.05-1**	**Jizan**	**Mexican lime (*Citrus* × *aurantiifolia*)**	**COI210501**	OP106898	**Cutp210501**	OP131622	**Wsp 210501**	OP131599<br>OP131605<br>OP131619
**21.05-3**	**Jizan**	**Mexican lime**	**COI210503**	OP106901	**Cutp210503**	OP131623	**Wsp 210503**	OP131604<br>OP131614
**21.05-4**	**Jizan**	**Mexican lime**	**COI210504**	OP106902	**Cutp210504**	OP131636	**Wsp 210504**	OP131598<br>OP131607<br>OP131615
**20.025-1**	**Al Baha**	**Sweet orange *(C. sinensis)***	**COI2002501**	OP106896	**Cutp2002501**	OP131624<br>OP131629<br>OP131639<br>OP131640	**Wsp 2002501**	OP131620
**20.025-3**	**Makkah**	**Sweet orange**	**-**	**-**	**Cutp2002503**	OP131627<br>OP131628<br>OP131632	**Wsp 2002503**	OP131602<br>OP131603<br>OP131606
**20.025-4**	**Najran**	**Sweet orange**	**COI2002504**	OP106903	**-**	-	**Wsp2002504**	OP131609
**20.025-5**	**Makkah**	**Sweet orange**	**COI2002505**	OP106893	**-**	-	**Wsp 2002505**	OP131613
**20.025-6**	**Makkah**	**Sweet orange**	**COI2002506**	OP106897	**Cutp2002506**	OP131625<br>OP131626	**Wsp 2002506**	OP131610
**20.025-7**	**Makkah**	**Mandarin *C. reticulata***	**COI2002507**	OP106899	**-**	-	**Wsp 2002507**	OP131611
**20.025-8**	**Najran**	**Mexican lime**	**COI2002508**	OP106904	**-**	-	**Wsp 2002508**	OP131612
**20.025-9**	**Al Baha**	**Sweet orange**	**COI2002509**	OP106900	**Cutp2002509**	OP131630<br>OP131633<br>OP131637	**Wsp 2002509**	OP131616

						OP131638		
**20.025-10**	**Makkah**	**Mandarin**	**COI2002510**	OP106905	**Cutp2002510**	OP131631	**Wsp 2002510**	OP131608<br>OP131617<br>OP131621
**Florida**	**Florida**	**Sweet orange**	**-**	OP106895<br>OP106894	-	OP131635<br>OP131634	**-**	OP131601<br>OP131600
