# Supplementary figures and images for: Characterization of the Asian Citrus Psyllid-‘Candidatus Liberibacter Asiaticus’ Pathosystem in Saudi Arabia Reveals Two Predominant CLas Lineages and One Asian Citrus Psyllid Vector Haplotype
Source: Microorganisms. 2022 Oct 8;10(10):1991. doi: 10.3390/microorganisms10101991 (PMC9610752; doi:10.3390/microorganisms10101991)

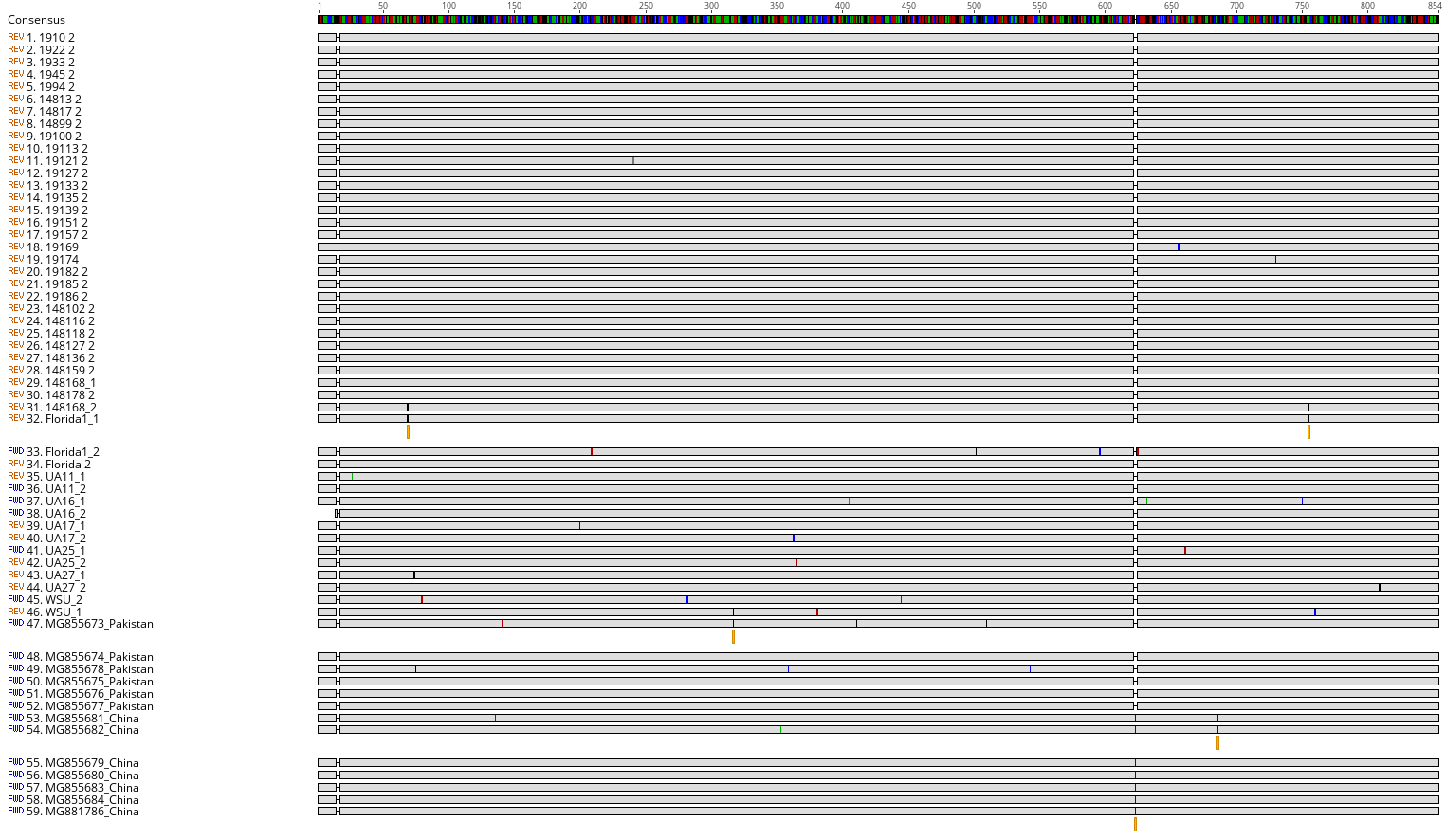

Supplement: Supplementary file 1 [file microorganisms-10-01991-s001.zip › Supplementary Figure S1.png]
